# Making non-compliance visible: how smart bins reshape social norms in urban waste management

**DOI:** 10.3389/fsoc.2026.1939810

**Published:** 2026-09-30

**Authors:** Claudio Marciano, Alessandro Sciullo, Filippo Barbera

**Affiliations:** 1 Claudio Marciano, Department of Political and International Sciences, University of Genoa, Genoa, Italy; 2 Department of Cultures, Politics and Society, Universita degli Studi di Torino, Turin, Italy

**Keywords:** science and technology studies, smart cities, social mechanism, social norms and community influence, waste management

## Abstract

Urban waste management increasingly relies on hybrid collection systems based on controlled-access smart bins. Their implicit promise is to strengthen compliance by increasing behavioural control while reducing the organisational costs associated with door-to-door collection. This article tests this premise through a survey of 502 users involved in a large-scale Italian pilot project. Integrating analytical sociology, social norms theory, and Science and Technology Studies, we identify three interrelated mechanisms underlying the system’s ambivalent outcomes: limited norm internalisation, ineffective sanctions, and the increased visibility of non-compliant behaviour. We argue that smart bins reshape users’ empirical expectations and may thereby contribute to the progressive erosion of compliance with waste separation norms.

## 1 Introduction

Urban waste management represents one of the most concrete testing grounds for the socioecological transition in European cities. At the level of policy objectives, major strategic documents show a significant degree of institutional convergence: from SDGs 11 and 12 (Ram et al., 2015), to the European Circular Economy Action Plan (Torres et al., 2020), and regional waste management plans (Marciano, 2023), a broadly shared vision has emerged, aimed at reducing overall waste generation, increasing separate collection, and promoting circular economy models.

Yet this convergence on ends is accompanied by considerable uncertainty regarding the means through which these goals should be pursued (Sabel and Victor, 2022). In waste management, one of the main governance challenges concerns the institutional and technological arrangements through which individual behaviour can be coordinated with the requirements of collection and treatment systems. Since individual disposal behaviour can only be partially monitored by service providers, establishing effective systems of monitoring and sanctioning becomes a central governance challenge (Coleman, 1990).

Since the 1990s, the progressive saturation of landfills and the growing environmental and economic benefits of recycling have encouraged the development of collection systems characterised by an increasing demand for behavioural control (Romano et al., 2021). This demand has been incorporated into a wide range of socio-technical devices, including collection calendars, differentiated containers, opening systems and disposal procedures (Marciano and Sciullo, 2025). Door-to-door collection represents the most explicit expression of this approach: by increasing the visibility of individual waste disposal, it makes behaviour more easily observable and therefore potentially sanctionable or incentivisable, as in *Pay-As-You-Throw* (PAYT) schemes (Romano and Masserini, 2023). Although this model has produced well-documented environmental benefits (Botti et al., 2020), it also entails substantial organisational costs, particularly in densely populated urban contexts (Massarutto, 2011). To overcome these limitations, hybrid collection systems based on controlled-access street containers, commonly referred to as *smart bins*, have become increasingly widespread (Gallardo et al., 2012). Their implicit premise is that more sophisticated technological devices can generate more effective norms and, consequently, higher levels of user compliance. This article puts that premise to the test.

The empirical analysis focuses on a hybrid collection system introduced in 2021 by Acea Pinerolese, a consortium managing waste collection services in 46 municipalities within the Metropolitan City of Turin. In part of the service area, traditional street collection was replaced by controlled-access containers operated through personal electronic cards, while the openings of residual waste containers were substantially reduced. During the first 3 years of implementation (2021–2024), the system produced an apparently contradictory outcome: overall waste generation—and particularly residual mixed waste—declined significantly, whereas the quality of separately collected materials progressively deteriorated.

How can this ambivalent outcome be explained? More specifically, to what extent did the new system mobilise users characterised by different interests, resources and motivational orientations? What role did expectations regarding sanctions and incentives play in shaping behavioural change? And how did the material configuration of the infrastructure influence the visibility of compliant and non-compliant behaviours?

We argue that this apparent contradiction results from the interaction of three interrelated social mechanisms. First, the limited capacity of the new service to mobilise users characterised by different interests, resources and motivational orientations. Second, the difficulty of translating the expectations of control generated by the infrastructure into effective systems of sanctioning and incentivisation. Third, the material configuration of smart bins, which makes noncompliant behaviours more visible than compliant ones, thereby reshaping users’ empirical expectations and progressively weakening the norms that sustain cooperation.

To address these questions, the article develops an interpretive framework integrating analytical sociology, as formalised in the Coleman Boat (Coleman, 1990; Ylikoski, 2011), with the theory of social norms (Bicchieri, 2006, 2016; Bicchieri et al., 2022), and the. This framework is operationalised through a survey administered to 502 users involved in the hybrid experiment. The survey data were analysed using k-means clustering, allowing the identification of four empirically grounded user profiles—Enthusiasts, Rule-Followers, Contractualists and Disengaged—representing distinct motivational orientations toward cooperation.

The article contributes to the literature in two respects. Empirically, it extends previous studies on social norms and waste management (Hewitt et al., 2023; Czajkowski et al., 2017) by shifting attention from information campaigns and normative feedback to the influence exerted by the socio-technical configuration of collection systems on user behaviour. Theoretically, it proposes and empirically tests an integration between analytical sociology and social norms theory, showing how socio-technical infrastructures shape not only opportunities for action but also the empirical and normative expectations that sustain cooperation. More broadly, the user typology proposed here may prove useful in analysing other public services whose effectiveness depends on sustained citizen cooperation.

The remainder of the article is organised as follows. Section 2 presents the theoretical framework. Section 3 describes the case study and the methodology. Section 4 discusses the empirical findings through the three logics of the Coleman Boat and introduces the proposed user typology. The final sections discuss the implications of the results and reflect on the opportunities and limitations of smart bins as instruments for governing environmental behaviour.

## 2 Theoretical framework

In ancient Rome, the *norma* was an L-shaped tool used by carpenters to draw straight lines and verify right angles. The regulatory function of this instrument has been preserved in the meaning the term subsequently acquired: a rule of conduct that individuals are expected—or believe they are expected—to follow in specific contexts, and whose violation is subject to sanctions (Opp, 2015).

Within the social sciences, the concept occupies a foundational position (Demeulenaere, 2011). Whereas rational choice theory takes individual interests—typically assumed to be utilitymaximising—as given and seeks to explain the functioning of social systems on that basis, normative theories tend to take norms as given and explain individual behaviour through them (Coleman, 1990).

Analytical sociology emerged as an attempt to bridge these two perspectives. Social phenomena are understood as the aggregated outcome of individual actions, but actors’ rationality is not conceived as the abstract maximisation of utility. Rather, it is contextually situated (Barbera and Negri, 2011) and depends on the “good reasons” that actors develop considering the constraints, opportunities and resources characterising the situations in which they are embedded (Boudon, 1981).

The Coleman Boat, the theoretical model through which Coleman formalised this perspective, summarises this approach through three fundamental logics (Ylikoski, 2011). The first is the logic of the situation, which describes how macro-social structures, institutions, and normative arrangements define the constraints and opportunities within which actors operate. The second is the logic of selection, which concerns the process through which actors, given the situation they face, choose among the available alternatives those they consider most consistent with their objectives and the resources at their disposal. The third is the logic of aggregation, which explains the mechanisms through which individual actions, by interacting with one another, generate collective outcomes that cannot be reduced to the simple sum of individual intentions (Boudon, 1981). Aggregate patterns emerge as properties of the system of interdependencies linking actors and explaining them requires reconstructing the mechanisms that connect the micro and macro levels.

The Coleman Boat provides a useful framework for explaining the emergence, maintenance, and erosion of social norms. Norms arise whenever individual actions generate externalities for others, creating a demand for behavioural control. For this demand to crystallise into an effective norm, however, beneficiaries must be able to establish sanctions that are both enforceable and sustainable (logic of the situation). Yet sanctions alone do not guarantee compliance. Especially where cooperation entails individual costs, actors develop different strategies of adaptation, ranging from full conformity to rule violation (logic of selection). Ultimately, even legally established norms depend on the beliefs individuals hold about the behaviour and expectations of others. In this respect, Bicchieri’s theory (Bicchieri, 2006, 2016) complements the Coleman Boat by interpreting social norms as systems of reciprocal empirical and normative expectations (logic of aggregation).

According to Bicchieri, compliance depends on two types of expectations. Empirical expectations refer to the belief that a sufficiently large proportion of individuals comply with a given norm. Normative expectations, by contrast, concern the belief that a sufficiently large proportion of individuals considers compliance appropriate and expects others to comply as well. The “others” to whom these expectations refer do not necessarily coincide with the population, but rather with specific reference networks—family members, friends, colleagues, neighbours, or social groups from whom individuals seek approval and recognition.

These expectations underpin not only the maintenance but also the erosion of social norms. A series of experimental studies (Bicchieri and Dimant, 2019; Bicchieri et al., 2022) has shown that observing norm violations tends to exert a stronger influence than observing compliance (Keizer et al., 2008): deviant behaviour modifies individuals’ empirical expectations more powerfully than compliant behaviour. This dynamic can trigger cumulative processes of normative erosion, particularly in cooperative settings and in the governance of commons. Subsequent research has shown that these processes may be mitigated by social proximity, namely the perception of belonging to socially meaningful groups. When individuals recognise themselves as members of the same reference community, compliant behaviours become more visible and influential, thereby reinforcing reciprocal expectations and increasing the resilience of social norms.

The new waste collection system introduced by Acea can be interpreted as a transformation of users’ action situation. The infrastructure redefines interests, resources, and capacities for control by introducing new rules and new material constraints (logic of the situation). Users respond by developing different strategies of adaptation, cooperation, resistance, or negotiation (logic of selection). The interaction among these strategies ultimately produces aggregate outcomes that depend crucially on the empirical and normative expectations formed and transformed over time (logic of aggregation).

While analytical sociology and social norms theory explain how individual behaviour generates collective outcomes, they pay less attention to the role played by material artefacts in shaping those behaviours. Since waste collection systems rely heavily on technological devices, these perspectives are complemented here with insights from Science and Technology Studies, which conceptualise artefacts as actors capable of mediating social action (Miller, 1987; Latour, 2005).

By recognising agency in artefacts and other non-human components of social processes, this perspective may appear to sit uneasily with the approaches discussed above, which assign a central role to actors’ intentionality. We address this apparent tension by incorporating STS insights within the analytical framework: artefacts contribute to defining the situation in which actors operate, enable or constrain programmes of action, and mediate the interactions through which aggregate outcomes emerge. In doing so, they may produce effects that go beyond the intentions of those who design, implement, and use them.

The normative content (Winner, 1980) embedded in smart bins can be summarised in two implicit messages. The first communicates that individual disposal behaviour may be observed and, if necessary, sanctioned. The second, conveyed through the restricted opening of the residual waste container, signals the need to reduce the disposal of this waste fraction.

In conveying these messages, smart bins act as intermediaries (Latour, 2005), translating the intentions of designers into material constraints that contribute to defining the logic of the situation. However, socio-technical artefacts do not simply transmit predefined programmes of action. They also mediate social interactions, generating interpretations, practices and unintended consequences that may diverge from the intentions of their designers (Volonté, 2017). In this sense, users do not merely comply with or resist technological prescriptions; they reinterpret, negotiate and sometimes transform them.

As the empirical analysis will show, the material properties of smart bins—controlled access through electronic cards, restricted openings and differentiated disposal procedures—gave rise to different strategies of adaptation (logic of selection). Among these was the possibility of circumventing the intended use of the system by leaving waste bags outside the containers. Paradoxically, the infrastructure thereby contributes to making non-compliant behaviours more publicly visible than compliant ones. This asymmetry, we argue, reshapes users’ empirical and normative expectations and constitutes one of the principal mechanisms through which the Acea experiment progressively lost its capacity to sustain cooperative behaviour.

## 3 Materials and methods

The theoretical framework was operationalised through an empirical research design structured in two main steps. The first consisted of administering a survey to users served by Acea Pinerolese’s new waste collection system. The second involved constructing a typology of users based on their responses, with the aim of identifying different orientations toward the new service and reconstructing the mechanisms linking the socio-technical configuration of the system to individual behaviour.

The questionnaire included 16 main questions, articulated into 58 items, together with four socio-demographic variables: housing type, household size, employment status, and residential area. The socio-demographic and housing variables were selected by the authors because they were considered particularly relevant to the practical opportunities and constraints under which household waste is produced, separated and disposed of. This choice was informed both by previous research showing that household composition, dwelling type and residential context are associated with differences in recycling and waste-sorting practices (Berger, 1997; du Toit and Wagner, 2020; Zhao and Chen, 2021), and by discussions with waste-management operators and sector experts conducted in the context of this and previous research projects. For instance, housing type and household size may influence the domestic space available for waste separation and temporary storage, while residential context may affect accessibility to collection points, opportunities for informal social monitoring, and the practical costs associated with disposal.

The analytical framework presented in Section 2 guided the selection of the questionnaire items. Of the 58 items included in the survey, 31 were used in the explanatory analysis because they directly operationalised the three logics of the Coleman Boat (Table 1). The remaining items addressed other aspects of the waste collection system that were part of the broader survey but were not directly related to the research questions addressed in this article, such as users’ satisfaction with specific components of the local waste service and difficulties encountered in sorting particular types of materials. The selection of the 31 items was therefore theoretically driven by the analytical framework rather than based on their empirical performance in the subsequent analysis.

**Table 1:** Empirical proxies for the three logics of action in the Coleman Boat.

Logic	Theoretical construct	Observed dimension	Item codes
Situation	Redefinition of interests, resources, technologies and control capacities	Information about the new service; understanding of decision-makers’ motivations; expectations regarding monitoring, pay-as-youthrow pricing, and sanctions	A1_1 A3_1 A3_2 A3_3 A3_4 A4_1 A5_1 A5_2 A5_3
Selection	Individual adaptation strategies	Changes in waste generation and sorting behaviour; household costs associated with the new service; operational difficulties	B10_1 B10_2 B10_3 B12_1 B12_2 B12_3 B13_5 B9_1 B9_2 B9_3
Aggregation	Formation of empirical and normative expectations	Discussion of the new service within social networks; observation of others’ behaviour and of the material traces of compliance and deviance	C14_1 C14_2 C14_3 C14_4 C14_5 C15_1 C15_2 C15_3 C15_4 C15_5 C15_6

The selected 31 items captured: (i) users’ understanding of the objectives and regulatory messages embedded in the new service (logic of the situation); (ii) the effects of the new system on household waste production, separation and disposal practices (logic of the selection); and (iii) users’ perceptions of the attitudes and behaviours of other citizens, including both discussion of the service within social networks and the observation of compliant and noncompliant behaviours (logic of the aggregation). The remaining items were used either for descriptive purposes or for profiling respondents.

All attitudinal variables were measured using five-point Likert scales ranging from 1 (“strongly disagree”) to 5 (“strongly agree”) (Likert, 1932). Before the main survey, the questionnaire was pilot-tested with several dozen users living in the study area to assess question clarity, wording and interview length. Minor revisions were introduced while preserving the theoretical structure of the instrument. The final survey was completed by a sample of 502 respondents, representative of a population of 61,429 residents across the eight municipalities included in the experiment. These municipalities were selected because they were the only ones in which the new service had been in operation for at least 2 years, a period considered sufficiently long to allow an analysis of recycling performance. The eight municipalities also present heterogeneous characteristics in terms of socio-demographic and economic variables.

The relationship between the aggregate outcomes and the survey data should be clarified. The administrative data on residual waste, separate collection and sorting quality describe macrolevel outcomes emerging from tens of thousands of individual disposal practices and their interdependencies within the socio-technical configuration of the service. The survey data are not used to establish a direct causal relationship between individual responses and these aggregate trends. Rather, they are used to reconstruct some of the social mechanisms that may have contributed to producing them.

Regarding the second step, user groups were therefore identified using the k-means clustering algorithm^1^ applied to the set of 31 items. Although the variables were derived from ordinal Likert scales, methodological research generally considers it acceptable to treat five-point Likert items as approximately continuous in exploratory analyses aimed at identifying response patterns rather than estimating causal relationships (Carifio and Perla, 2008; Norman, 2010; Sullivan and Artino, 2013).

User groups were identified using the k-means clustering algorithm applied to the set of variables representing the three logics of the Coleman Boat. Solutions ranging from two to six clusters were explored. For each solution, clustering quality was assessed using the WithinCluster Sum of Squares (Inertia), the Silhouette coefficient (Rousseeuw, 1987), the Calinski–Harabasz index (Caliński and Harabasz, 1974), and the Davies–Bouldin index (Davies and Bouldin, 1979). Although the variables were derived from ordinal Likert scales, methodological research generally considers it acceptable to treat five-point Likert items as approximately continuous in exploratory analyses aimed at identifying response patterns rather than estimating causal relationships (Carifio and Perla, 2008; Norman, 2010; Sullivan and Artino, 2013). On this basis, the k-means algorithm was deemed appropriate.

## 4 Results

This section presents the main findings of the analysis according to the logics of action proposed by Coleman. Four users profiles, corresponding to as many models of action, are identified (Section 4.1) and they are then examined in terms of the macro–micro (Section 4.2), micro–micro (Section 4.3), and micro–macro (Section 4.4) dynamics generated by the new hybrid waste collection system.

Before presenting the findings, it is useful to recall the aggregate outcome that the analysis seeks to explain. On the one hand, the new collection system substantially reduced overall waste generation, particularly residual mixed waste, while increasing separate collection, although the increase was not proportional to the reduction in residual waste. On the other hand, the quality of the collected materials progressively deteriorated. Sorting facilities reported increasing levels of contamination in recyclable fractions—especially paper and plastics—indicating a growing incidence of improper waste sorting.

### 4.1 User profiles: four motivational orientations

The cluster analysis performed on the 31 items referred to the three logics, identified four user types, corresponding to four distinct models of action emerging from the joint consideration of individual, interactional and contextual factors that jointly build the normative environment represented by the 31 items considered for the cluster analysis. In the next sections a deep analysis of how the different models of action work along the three logics is provided while the main characteristics of each cluster are reported in Figure 1.

**Figure 1:**
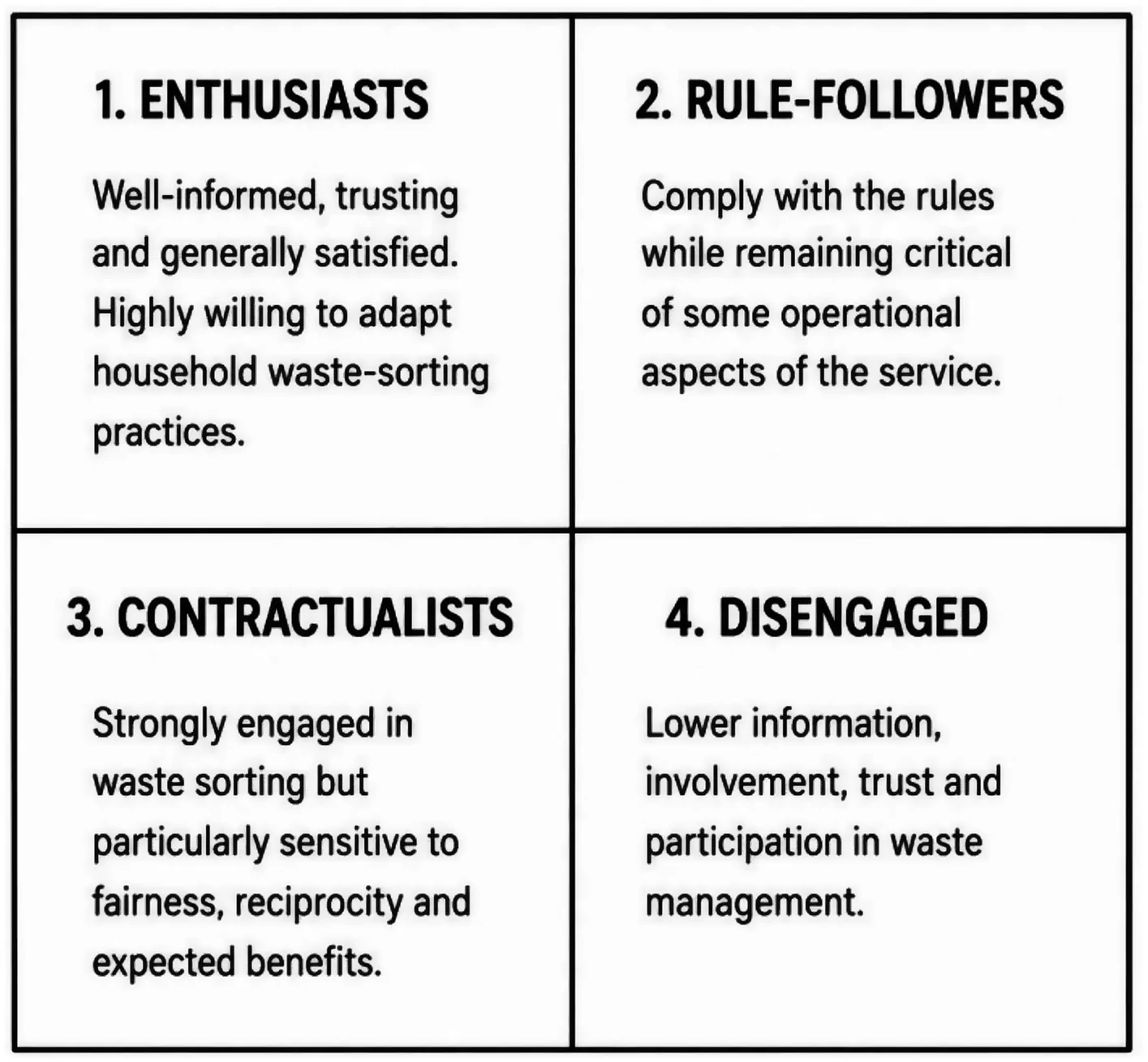
Short description of clusters.

The Enthusiasts are well-informed, trusting users who are generally satisfied with the service. They show a strong willingness to adapt their household practices to the objectives of the new system and view waste separation as consistent with environmental values they had already internalised. Demographically, this cluster is characterised by a relatively older average age, a predominance of employees and retirees, smaller households (single occupants and couples), and detached housing.

The Rule-followers comply with the rules while maintaining a more critical attitude towards some organisational aspects of the service. The new collection system appears to have had only a limited impact on their household waste sorting practices. Their compliance seems to be motivated primarily by the perceived legitimacy of public authority. Members of this cluster mainly live in single- or multi-family houses outside urban centres and are predominantly employees or economically inactive respondents.

The Contractualists show high levels of engagement in waste sorting practices but make their willingness to cooperate conditional upon the perceived fairness, reciprocity, and effectiveness of the system. Their compliance largely depends on the credibility of the rules, the existence of tangible benefits, and the behaviour of other users. This cluster includes a slightly higher proportion of women, apartment dwellers, and respondents outside the labour market.

Finally, the Disengaged are characterised by lower levels of information, involvement, and interest in waste management. In the absence of strong environmental values or institutional motivations, their behaviour appears to be more strongly influenced by contextual cues and by expectations regarding the behaviour of other users. Members of this cluster predominantly live in urban areas, include relatively large proportions of students, homemakers, and selfemployed professionals, and are more likely to belong to larger households.

These four clusters represent different ways of interpreting the norms embedded in the new collection system. The same socio-technical infrastructure therefore produces different effects depending on users’ motivations, expectations, and sources of normative legitimacy. In this respect, the typology also resonates with insights from pragmatic sociology on the plurality of evaluative grammars through which actors interpret and justify environmental action (Blok, 2013). The four profiles should not be understood as corresponding to distinct orders of worth, but they show that cooperation around environmental infrastructures may be supported by different principles of evaluation, ranging from environmental commitment and institutional legitimacy to fairness and reciprocity.

### 4.2 Logic of the situation

The introduction of Acea’s new collection system constitutes an example of a new *action situation*. This new situation was created by the decision-makers—public administrators and Acea managers. Although they control key resources, such as the financial and organisational levers of the service, decision-makers do not directly control users’ behaviour, which is essential for achieving their objectives of reducing residual waste and improving the quality of separate collection. Through the new rules, to use Coleman’s terminology, they generate a “demand for the transfer of control rights,” expressed primarily through controlled access to the containers and their redesigned physical configuration.

The survey results provide insight into how users interpreted and responded to this new action situation (Table 2).

**Table 2:** Mean scores of the items proxy of the logic of the situation, by cluster.

Items	Enthusiasts	Rule-followers	Contractualists	Disengaged
A1_1 I received sufficient information about the new service	4.267	4.319	4.316	2.893
A1_2 I have all the information I need to comply with the rules	4.473	4.327	4.406	3.388
A2_1 The reasons behind the introduction of the new service are clear to me	3.612	3.700	3.905	1.725
A2_2 The new service was introduced to improve the quality of separate collection	4.252	3.531	3.677	2.029
A2_3 The new service was introduced to reduce environmental impacts	4.344	3.460	4.232	2.204
A2_4 The new service was introduced to reduce costs	3.542	2.938	2.987	1.796
A3_1 I expect to pay according to the amount of waste I dispose of	3.931	3.761	3.839	2.757
A3_2 I expect the electronic card to improve separate collection	4.382	4.301	4.600	3.126
A3_3 I expect the electronic card to improve monitoring	4.229	4.195	4.471	3.000
A3_4 I am concerned about receiving sanctions if I do not sort my waste correctly	3.076	2.673	3.490	2.311

A first noteworthy finding concerns the gap between users’ perceived level of information and their understanding of the decision-makers’ motivations. With the exception of the Disengaged, who reported being poorly informed, most respondents considered the information they had received about the functioning of the new system to be sufficient (A1_1) and believed they possessed the knowledge required to comply with its rules (A1_2). Mean scores declined substantially, however, when respondents were asked to evaluate the reasons underlying the introduction of the new service. Environmental motivations—improving the quality of separate collection (A2_2) and reducing environmental impacts (A2_3)—received consistently higher levels of agreement than economic motivations (A2_4), which obtained lower scores across all clusters.

A second finding concerns the expectations generated by the new action situation. Users recognised the system’s potential capacity to monitor and trace individual disposal behaviour. Expectations regarding a future link between the quantity of waste disposed of and waste charges (A3_1) were relatively high, as were expectations that the electronic access card would improve monitoring (A3_3) and the quality of separate collection (A3_2). By contrast, concern about receiving sanctions for incorrect waste sorting (A3_4) was substantially weaker. This finding is consistent with the institutional design of the pilot project, which introduced neither new sanctioning mechanisms nor explicit pay-as-you-throw pricing. Users therefore perceived an increase in the system’s monitoring capacity without believing that this would translate into immediate consequences for their own behaviour.

Overall, perceptions of the new action situation were remarkably similar across the different user groups, with the notable exception of the Disengaged, who reported consistently lower evaluations on virtually all dimensions. Some differences nevertheless emerged. Enthusiasts expressed the highest overall expectations regarding the new system, Contractualists were more convinced of its environmental rationale, whereas Rule-followers expressed stronger expectations concerning the future introduction of pay-as-you-throw pricing.

### 4.3 Logic of selection

The logic of selection concerns the way actors choose among the different courses of action made possible by the situation. In other words, it refers to the process through which individuals. Through which individuals take a course of action among the available alternatives (e.g., doing a proper separation of waste or not) or on the basis of their interests, resources, perception and compliance with social and formal norms.

Although embedded in technological devices and not supported by explicit sanctioning mechanisms, the rules governing the new collection system are first and foremost legal rules. Proper waste sorting and the correct use of collection containers are mandatory obligations rather than optional forms of cooperation. Individual users may therefore adopt behaviours that do not align with the objectives of the decision-makers. Such non-compliance, however, is unlikely to take the form of open disobedience; rather, it is more likely to occur in the “backstage” of social life (Goffman, 1959), beyond the reach of direct observation.

The survey nevertheless provides insight into how users describe their own behaviour within this backstage. Section B of the questionnaire explores the ways in which respondents produce, separate, and dispose of household waste (Table 3).

**Table 3:** Mean scores of the items proxy of the logic of selection, by cluster.

Items	Enthusiasts	Rule-followers	Contractualists	Disengaged
B1_1 I have noticed that I produce more waste	1.847	1.274	2.413	1.602
B1_2 I have noticed that I produce more separately collected waste	3.099	1.345	3.813	1.961
B1_3 I have noticed that I produce more residual mixed waste	2.168	1.221	2.652	1.883
B2_1 I pay greater attention to waste separation	4.588	1.814	4.523	3.126
B2_2 I have reorganised household space to facilitate waste separation	4.267	1.460	4.381	2.456
B2_3 I pay greater attention to what I dispose of as residual mixed waste	4.481	1.478	4.639	2.767
B3_1 I find it difficult to remember to bring the electronic card	1.412	1.938	2.039	2.427
B3_2 The container openings are inconvenient to use	3.008	3.796	3.910	4.252
B3_3 The areas around the containers are less clean	2.702	3.310	3.735	3.845

The four clusters further accentuate the differences in users’ behavioural strategies.

The Enthusiasts once again emerge as the group most willing to align their behaviour with the objectives pursued by the public authorities. They report the lowest levels of inconvenience associated with the electronic card and the restricted openings of the containers (items B3), and they are also the group least likely to perceive waste abandonment around collection points as a serious problem. Moreover, they obtain the highest scores on items capturing greater commitment to waste separation and increased efforts to reduce residual waste.

The Rule-followers, by contrast, display a distinctly different profile. They are more critical of the restricted container openings and of the cleanliness of the areas surrounding the collection points, while reporting relatively limited changes in their own waste production and disposal practices. Their evaluation suggests that the new service increases the costs borne by users without substantially influencing their own behaviour. Although they declare themselves compliant, they do not regard the new rules as particularly effective in changing behaviour.

The Contractualists and the Disengaged once again appear as the groups least willing to transfer their resources without reservation and those most inclined to criticise the new service. Both assign relatively high scores to indicators of dissatisfaction, such as the inconvenience of the containers and the poor cleanliness of the surrounding areas. Although the electronic access card is generally not perceived as highly problematic, these respondents nevertheless regard it as unnecessarily inconvenient and argue that it should allow users to access all containers throughout the municipality.

The trajectories of these two groups diverge, however, when considering waste production and sorting practices. The Contractualists report even stronger behavioural adjustments than the Enthusiasts, declaring greater attention to waste separation, more extensive reorganisation of household space, and increased care in reducing the residual waste fraction. This finding suggests an important dynamic: users who are critical of the service while simultaneously investing considerable effort in complying with its requirements. Their implicit message to the public administration appears to be that they have made a substantial investment in cooperation and therefore expect corresponding improvements in service quality or institutional recognition. This contractual orientation is entirely absent among the Disengaged, whose relationship with the service is characterised less by active opposition than by weak integration into the normative logic of the collection system. These users are both critical of the service and relatively detached from waste management as a public issue, showing little interest in engaging with the public authorities.

### 4.4 Logic of aggregation

The third dimension of the explanation concerns the logic of aggregation, namely the microto-macro process through which individual users’ actions interact to generate relatively stable collective outcomes. The objective is to identify the social mechanisms through which users’ perceptions and behaviours mutually influence one another, gradually shaping the overall functioning of the waste collection system.

In the present case, these interactions operate primarily through two distinct forms of expectations corresponding to Bicchieri’s framework (Table 4). Section C1 explores users’ perceptions of how the new service is evaluated within their social networks—that is, the conversations among relatives, friends, and acquaintances through which shared interpretations of the new rules and their purposes are formed. Section C2 focuses instead on users’ direct or indirect observation of other people’s behaviour, either during waste disposal or through the material traces these behaviours leave in public spaces.

**Table 4:** Mean scores of the items proxy of the logic of selection, by cluster.

Items	Enthusiasts	Rule-followers	Contractualists	Disengaged
C1_1 The new service was widely discussed when it was introduced	3.069	3.726	4.148	2.563
C1_2 The new service is still discussed fairly often	2.366	2.805	3.490	2.505
C1_3 My friends and relatives are satisfied with the new service	3.489	2.894	3.471	1.864
C1_4 My friends and relatives hold very different opinions about the new service	2.916	3.345	3.394	2.447
C2_1 Some people dispose of their waste outside their municipality of residence	1.603	1.549	1.974	1.650
C2_2 People in my neighbourhood comply with the rules	3.763	3.381	3.426	3.039
C2_3 Compliance with the rules varies across neighbourhoods	3.359	3.345	3.858	3.049
C2_4 Many people dispose of their waste in other municipalities	1.916	2.204	3.245	2.068
C2_5 Many people mix waste into the recyclable fractions	2.359	2.876	3.897	2.689
C2_6 People in my apartment building comply with the rules	3.809	3.894	4.148	3.767
C2_7 Waste abandonment around collection points has increased	2.603	3.372	3.948	3.961

The diversity of opinions surrounding the new service (C1) suggests that the organisational norms embedded in the new collection system have not achieved widespread legitimacy. Although respondents do not necessarily question the value of separate collection itself, they perceive considerable disagreement regarding the specific ways in which it is organised and implemented.

The four clusters differ substantially in this respect. Enthusiasts report predominantly positive evaluations of the service among friends and relatives, whereas Disengaged users appear to belong to social networks where attitudes are generally more critical, a pattern also observed among Contractualists.

As discussed in Section 2, research on social norms shows that empirical expectations play a decisive role in shaping compliance and norm erosion. Moreover, observing norm violations has a stronger influence on behaviour than observing compliance, particularly in the management of commons, where cooperative behaviour requires individuals to incur personal costs (Bicchieri et al., 2022).

From this perspective, the findings reported in Section C2 are particularly revealing. The variables measure users’ perceptions of the most common forms of rule violation. The behaviours perceived as most widespread are the abandonment of waste bags near collection points and the improper sorting of recyclable materials. It is noteworthy, however, that none of the clusters reported these behaviours as being particularly common within their own neighbourhoods or residential buildings.

The Contractualists stand out most clearly from the other groups in the frequency with which they perceive opportunistic behaviour and rule violations. Although they report the strongest commitment to waste separation, they are also the group most likely to observe waste disposal outside municipal boundaries, incorrect sorting practices, and waste abandonment (C2_3–C2_5). This finding is consistent with a pattern of conditional cooperation, in which the willingness to bear the costs of proper waste separation depends crucially on the perception that other users are also contributing fairly to the collective effort.

## 5 Discussion

Why did Acea’s users substantially reduce overall waste generation, particularly the residual mixed waste fraction? Why this reduction is only partially balanced by a correspondent increase in separated waste? And why, despite this reduction, did the quality of separated waste progressively deteriorate, especially with respect to plastics, paper, cardboard, and other recyclable fractions?

The survey points to three interrelated explanatory mechanisms: first, *technological budging* does not foster the internalisation of norms and therefore produces only limited changes in users’ attitudes; second, norms that are not supported by effective positive or negative sanctions fail to sustain compliant behaviour; third, infrastructures that increase the visibility of deviant behaviour, without making compliant behaviour equally visible, facilitate the erosion of social norms. This section discusses these three mechanisms in relation to the theoretical framework presented above and to the existing literature on social norms and waste management.

Budging refers to the incorporation of insights from behavioural sciences into legal and regulatory instruments governing domains in which cooperation is mandatory (Oliver, 2013). Unlike the better-known concept of nudging (Thaler and Sunstein, 2008), budging relies on coercive interventions that do not allow users to opt out. To discourage smoking, for example, a nudge consists of warning messages printed on cigarette packages, whereas a budge would be represented by smoke detectors that trigger a fire alarm when someone lights a cigarette in the restroom of a train.

The waste collection system implemented by Acea belongs to this second category. The electronic card is mandatory, and containers cannot be opened without it. Yet, like nudges, budges do not seek to modify individuals’ moral norms. They influence behaviour without changing underlying attitudes. They facilitate or constrain actions, but they do not persuade.

The survey findings clearly illustrate the limits of this approach. Decision-makers succeeded in eliciting cooperative behaviour from users who were already predisposed to cooperate—albeit for different reasons—such as the Enthusiasts and the Rule-followers. However, they failed to alter the “good reasons” underlying the behaviour of the Contractualists, whose demand for reciprocity through positive incentives remained unanswered, or of the Disengaged, who remained largely detached from the normative logic of the system.

This observation leads directly to the second mechanism, which is well established in the literature on social norms: rules unsupported by effective sanctions tend to encourage noncompliant behaviour. Coleman (1990) described this as the problem of the second-order free rider: a norm can only be effective if the sanctions sustaining it are themselves enforceable at reasonable cost. The design of the smart bins implicitly promises stronger monitoring and the potential introduction of positive incentives, such as pay-as-you-throw pricing. Survey results indicate that users clearly recognised this promise. Expectations regarding the future use of the electronic card for monitoring waste disposal and implementing variable pricing are consistently high across all user groups. Yet these remain expectations about future possibilities rather than present realities, as demonstrated by the low perceived likelihood of sanctions reported by all clusters, including the Disengaged. Users’ perceptions accurately reflect the institutional reality. Smart bins promise sanctions that they cannot deliver. While the electronic card records the number of disposals, it does not verify whether waste has been correctly sorted. Three years after the beginning of the pilot project, none of the municipalities served by Acea had introduced pay-as-you-throw pricing and no municipality using smart bins has successfully implemented such a system. Circumventing the rules remains straightforward: users can simply leave waste bags beside the containers or dispose of them in neighbouring bins that do not require controlled access.

The third mechanism follows directly from the second. Making deviant behaviour more visible contributes to the erosion of social norms, particularly when compliant behaviour remains largely invisible. Research on social norms has consistently shown that observing violations influences empirical expectations more strongly than observing compliance (Bicchieri and Dimant, 2019; Keizer et al., 2008). In the case of smart bins, this psychological asymmetry is reinforced by the material characteristics of the infrastructure itself. Noncompliant behaviour leaves highly visible traces in public space—waste bags abandoned beside containers or improperly sorted materials that are readily observable—whereas compliant behaviour disappears once waste has been correctly deposited.

The survey findings support this interpretation. The two groups most exposed to normative erosion—the Contractualists and the Disengaged—are also those reporting the highest frequency of opportunistic behaviours, including waste abandonment, disposal in neighbouring municipalities, and incorrect sorting. Among Contractualists these perceptions generate a form of structural frustration. Users who invest considerable effort in proper waste sorting repeatedly observe rule violations without perceiving any institutional response. Their conditional compliance is therefore undermined not by a lack of motivation, but by the gradual erosion of the empirical expectations on which cooperation depends.

These three mechanisms also help explain the temporal dynamics observed in the case study. During the initial phase of the pilot project, the new service primarily operated through the logic of the situation: the material constraints introduced by the infrastructure rapidly reduced the disposal of residual waste across all user groups. Over time, however, the failure to implement the sanctions implicitly promised by the system’s design, together with the increasing visibility of rule violations, progressively reshaped users’ empirical expectations. This erosive process affected the four user profiles differently. Enthusiasts and Rule-followers, whose behaviour was supported either by pre-existing environmental values or by respect for institutional authority, proved relatively resilient. Contractualists, whose cooperation depended on reciprocity and institutional credibility, became increasingly vulnerable to normative erosion. Disengaged users, by contrast, remained weakly integrated into the normative logic of the service throughout the period of observation.

These findings are broadly consistent with the experimental literature on waste management. Hewitt et al. (2023) showed that regularly communicating recycling performance at the condominium level increases recycling rates only when combined with comparisons with the average performance of similar communities, thereby activating mechanisms of social proximity. Likewise, Czajkowski et al. (2017), analysing approximately 1,800 households in three Polish cities, found that individuals’ willingness to bear the costs of separate collection depends strongly on the perception that such behaviour is widespread and socially approved. In both cases, the contextual dimension of social norms—the perception that cooperation is prevalent within one’s reference group—emerges as a decisive factor that infrastructure alone cannot generate.

The Acea case contributes an additional insight. The salience of empirical and normative expectations depends not only on communication or institutional arrangements but also on the material configuration of the infrastructure itself. Compared with door-to-door collection, smart bins do not substantially increase the traceability of individual waste disposal to make incentives or sanctions operational. Instead, they increase the visibility of non-compliant behaviour, amplifying the structural asymmetry that leads users to overestimate the prevalence of rule violations. Compared with conventional street collection, smart bins add the promise of greater control without delivering its consequences, while lacking the social proximity that characterises door-to-door systems, where residents can directly observe neighbours engaging in appropriate waste sorting practices.

In summary, the three mechanisms taken together can be understood as a complex process unfolding across the three logics of the Coleman Boat. At the level of the logic of the situation, the introduction of controlled-access containers and restricted openings redefined users’ opportunities, constraints, and expectations by increasing the perceived traceability of waste disposal and signaling the possible introduction of sanctions or pay-as-you-throw pricing. At the level of the logic of selection, users responded to this new setting in different ways according to their motivations and resources. While some adapted because of environmental values or respect for institutional rules, others made their compliance conditional on reciprocity, fairness, and credible enforcement, and still others remained weakly engaged. Finally, at the level of the logic of aggregation, these heterogeneous responses interacted over time through users’ observations of one another. Because non-compliant behaviour left visible traces in public space while compliant behaviour remained largely invisible, empirical expectations of widespread cooperation gradually weakened, particularly among conditional and disengaged users. The result of the complex interaction among the three mechanisms over the three logics is a cumulative process in which technological constraints continued to influence disposal practices, while the normative foundations of cooperation gradually eroded. The ambivalent outcome of the Acea experiment therefore reflects not a simple failure of technology, but a misalignment between infrastructural design, institutional enforcement, and the social expectations required to sustain collective compliance.

## 6 Conclusion

This article has examined the implementation of a controlled-access street collection system introduced by Acea Pinerolese through a theoretical framework integrating analytical sociology and social norms theory. The case study shows that the new system produced an apparently paradoxical outcome: a substantial reduction in residual mixed waste accompanied by a progressive deterioration in the quality of separate collection. The analysis, based on the Coleman Boat and on the identification of four user profiles, suggests that this ambivalent outcome cannot be explained solely by the technical characteristics of the infrastructure. Rather, it reflects the ways in which the socio-technical configuration reshapes users’ interests, expectations, and strategies of cooperation.

The first is practical and transformative, in the sense attributed by the pragmatist tradition in social research (Prasad, 2022), as it offers insights for the design of waste collection systems. The findings suggest that smart bins should not be regarded as an organisational shortcut to door-to-door collection, particularly in contexts where separate collection is not yet firmly established and user cooperation remains fragile. Under such conditions, the limited capacity of smart bins to enforce effective monitoring and sanctions, combined with the increased visibility of opportunistic behaviour, may gradually weaken cooperative norms. Conversely, these systems may prove more effective in contexts where waste separation has already been widely internalised and where cooperative user profiles, such as the Enthusiasts and Rulefollowers identified in this study, predominate.

In the Acea Pinerolese case, smart bins were adopted as an alternative to door-to-door collection mainly because of the higher labour costs and organisational complexity associated with the latter. To the authors’ knowledge, however, this choice was not supported by a comparative assessment of the medium- and long-term costs and benefits of the two systems. While door-to-door collection generally entails higher labour costs, it may require lower investment in technological infrastructure and collection equipment and generate higher revenues from better-quality recyclable materials. A meaningful comparison should therefore consider the overall performance of the two systems over time rather than their immediate operating costs alone.

This study, which originated from the local decision-makers interest in assessing the strengths and weaknesses of the system they had introduced, may provide a basis for such organisational learning. One possible strategy would be to pilot door-to-door collection in a small number of “sentinel” municipalities—including Pinerolo and municipalities representing different territorial characteristics—and compare its performance with the existing system across waste fractions and over time. Such experimentation might initially entail higher costs and organisational difficulties, but it would allow decision-makers to assess whether these costs are compensated in the medium term by improved collection quality, higher material recovery, and more stable user cooperation.

More generally, smart bins appear to prioritise the logistical and organisational requirements of waste collection—greater investment in technological capital, service rationalisation, and reductions in operational and labour costs—over the objective of fostering lasting behavioural change. Public administrations considering the adoption of these systems should therefore clearly communicate the objectives they intend to pursue, avoiding unrealistic expectations regarding pay-as-you-throw pricing or the effective monitoring of individual waste disposal, which remain difficult to implement under current technological and organisational conditions. At the same time, service design should recognise the diversity of users’ motivational orientations and complement technological devices with strategies aimed at strengthening empirical expectations favourable to cooperation. This requires investment not only in the initial introduction of the service but also throughout its implementation, involving intermediary organisations, neighbourhood associations, condominium managers, and other local opinion leaders in continuous processes of communication, feedback, and collective learning.

The second contribution is theoretical. The study shows that integrating analytical sociology with social norms theory provides a promising framework for understanding waste management systems, an area in which this conceptual approach has so far been only marginally applied. Combining Coleman and Bicchieri makes it possible to explain not only how infrastructures reshape users’ interests, constraints, and opportunities, but also how they contribute to the formation of the empirical and normative expectations that sustain cooperation. From this perspective, smart bins emerge not merely as technological or organisational devices but as normative infrastructures actively involved in the creation, maintenance, and erosion of social norms.

More broadly, this article contributes to a perspective that considers technological change an important driver of socio-ecological transitions, but not an independent variable. At each stage of innovation—from the definition of needs to technological design and implementation—technologies interact with institutional arrangements, social norms, and the characteristics of their contexts of application. This calls not only for greater integration between technical and social-scientific knowledge, but also for the involvement of social scientists beyond the expost evaluation and critique of innovation. Sociological knowledge can contribute upstream to the selection and design of technologies and to the organisational arrangements through which they are implemented.

The Acea case illustrates the relevance of such an approach. The design of smart bins and their implementation in the Pinerolese context were primarily driven by considerations of technical functionality, control capacity, and organisational efficiency, while less attention was devoted to how these devices would interact with heterogeneous users, existing social norms, and local practices. The problem was therefore not simply that the technology failed to produce some of its expected outcomes. Rather, these social dimensions could have been incorporated into the design and implementation process itself, potentially informing the choice of technological components, operating procedures, monitoring arrangements, and complementary forms of user engagement.

Finally, the mechanism-based and actor-centred perspective adopted here does not exhaust the possible ways of analysing the interaction between material infrastructures and waste-sorting behaviour. Future research could complement this framework with social practice theory (Shove et al., 2012), shifting attention from individual orientations and expectations to the ways in which technological change reconfigures the materials, competences and meanings that constitute routinised waste-sorting practices. The survey used in this study was not designed to operationalise these dimensions systematically, but their integration represents a promising direction for further research.

## Data Availability

The raw data supporting the conclusions of this article will be made available by the authors, without undue reservation.

## Footnotes

1 For clustering procedures the Python scikit-learn library was adopted (Pedregosa et al., 2011). Solutions ranging from two to six clusters were explored. For each solution, clustering quality was assessed using the Within-Cluster Sum of Squares (Inertia), the Silhouette coefficient (Rousseeuw, 1987), the Calinski–Harabasz index (Caliński and Harabasz, 1974), and the Davies–Bouldin index (Davies and Bouldin, 1979).
